# Alcohol Consumption in Diabetic Patients with Nonalcoholic Fatty Liver Disease

**DOI:** 10.1155/2017/7927685

**Published:** 2017-11-01

**Authors:** Preya J. Patel, David Smith, Jason P. Connor, Leigh U. Horsfall, Kelly L. Hayward, Fabrina Hossain, Suzanne Williams, Tracey Johnson, Katherine A. Stuart, Nigel N. Brown, Nivene Saad, Andrew D. Clouston, Katharine M. Irvine, Anthony W. Russell, Patricia C. Valery, Elizabeth E. Powell

**Affiliations:** ^1^Department of Gastroenterology and Hepatology, Princess Alexandra Hospital, Brisbane, QLD, Australia; ^2^Centre for Liver Disease Research, Translational Research Institute, School of Medicine, The University of Queensland, Brisbane, QLD, Australia; ^3^QIMR Berghofer Medical Research Institute, Brisbane, QLD, Australia; ^4^Discipline of Psychiatry, The University of Queensland, Brisbane, QLD, Australia; ^5^Inala Primary Care, Brisbane, QLD, Australia; ^6^Pathology Queensland, Brisbane, QLD, Australia; ^7^Department of Radiology, Princess Alexandra Hospital, Brisbane, QLD, Australia; ^8^Mater Research, Translational Research Institute, University of Queensland, Brisbane, QLD, Australia; ^9^School of Clinical Medicine, The University of Queensland, Brisbane, QLD, Australia; ^10^Department of Diabetes and Endocrinology, Princess Alexandra Hospital, Brisbane, QLD, Australia

## Abstract

**Aim:**

To examine the association between lifetime alcohol consumption and significant liver disease in type 2 diabetic patients with NAFLD.

**Methods:**

A cross-sectional study assessing 151 patients with NAFLD at risk of clinically significant liver disease. NAFLD fibrosis severity was classified by transient elastography; liver stiffness measurements ≥8.2 kPa defined significant fibrosis. Lifetime drinking history classified patients into nondrinkers, light drinkers (always ≤20 g/day), and moderate drinkers (any period with intake >20 g/day).

**Result:**

Compared with lifetime nondrinkers, light and moderate drinkers were more likely to be male (*p* = 0.008) and to be Caucasian (*p* = 0.007) and to have a history of cigarette smoking (*p* = 0.000), obstructive sleep apnea (*p* = 0.003), and self-reported depression (*p* = 0.003). Moderate drinkers required ≥3 hypoglycemic agents to maintain diabetic control (*p* = 0.041) and fibrate medication to lower blood triglyceride levels (*p* = 0.044). Compared to lifetime nondrinkers, light drinkers had 1.79 (95% CI: 0.67–4.82; *p* = 0.247) and moderate drinkers had 0.91 (95% CI: 0.27–3.10; *p* = 0.881) times the odds of having liver stiffness measurements ≥8.2 kPa (adjusted for age, gender, and body mass index).

**Conclusions:**

In diabetic patients with NAFLD, light or moderate lifetime alcohol consumption was not significantly associated with liver fibrosis. The impact of lifetime alcohol intake on fibrosis progression and diabetic comorbidities, in particular obstructive sleep apnea and hypertriglyceridemia, requires further investigation.

## 1. Introduction

In people with type 2 diabetes mellitus (T2DM), modest alcohol intake is associated with a lower risk of cardiovascular disease and mortality. Analysis of a large T2DM cohort (*n* = 11,140; 30% drinkers) followed up for five years found that, compared with abstainers, moderate alcohol consumption (≤21 and ≤14 standard drinks weekly for men and women, resp.) was associated with a 17% lower risk of cardiovascular events, a 15% lower risk of microvascular complications, and a 13% lower risk of all-cause mortality [1]. These cardiovascular benefits may be a consequence of enhanced insulin sensitivity and favorable effects on inflammation and high-density lipoprotein (HDL) levels [2]. In contrast, higher levels of alcohol intake were associated with increased cardiovascular events and all-cause mortality in a dose-dependent manner [1].

Limited data exist, however, regarding the effect of alcohol use on other diabetic complications. In particular, people with T2DM have a high prevalence of nonalcoholic fatty liver disease (NAFLD), are more likely to develop cirrhosis, and have a 2-3-fold increased risk of mortality from chronic liver disease [3, 4]. It is therefore important to determine whether alcohol intake influences liver disease development or progression in diabetic patients with NAFLD. By definition, a diagnosis of NAFLD requires the exclusion of significant alcohol consumption in patients with steatosis and metabolic risk factors [5]. Population studies have shown a dose-response relationship between the level of alcohol use and the risk of liver injury, with an increase in relative risk observed at approximately 1-2 and 2-3 standard drinks per day for women and men, respectively [6–8]. The effect of modest alcohol intake in patients with NAFLD remains controversial, with some studies suggesting that low-to-moderate alcohol consumption is associated with lesser NAFLD severity [9–11]. However, in these studies, the prevalence of patients with T2DM or metabolic syndrome was relatively low, ranging from 7% to 41% of individuals within each study cohort [9–12].

In diabetic patients with NAFLD who have an increased risk of nonalcoholic steatohepatitis (NASH) and fibrosis, the effect of modest alcohol consumption on fibrosis progression and patient outcomes remains unclear. In order to investigate this, we examined the association between lifetime alcohol consumption and liver stiffness, a noninvasive marker of liver fibrosis, in a cohort of diabetic patients at risk of clinically significant NAFLD. Additionally, we examined the association between alcohol consumption and other comorbidities and medication use.

## 2. Methods

Consecutive patients with T2DM were offered participation in a cross-sectional study when they attended the Endocrine Clinic at the Princess Alexandra Hospital, Brisbane (*n* = 96), or from primary care practices within the Metro South Hospital and Health Services District (*n* = 55), between October 2015 and November 2016.

### 2.1. Case Ascertainment/Study Eligibility

Patients were eligible to be included in the study if they had a diagnosis of T2DM defined using standard criteria [13], NAFLD, and an indeterminate or high score on the FIB-4 [14] or NAFLD fibrosis scores [15]. A diagnosis of NAFLD required demonstration of hepatic steatosis by liver ultrasound in the presence of metabolic risk factors and the exclusion of significant alcohol consumption (>20 g/day) within the preceding 5 years, steatosis-inducing drugs, or other chronic liver diseases (including a prior history of alcohol-related liver disease). Patients were excluded if they had stage 5 chronic kidney disease (estimated glomerular filtration rate <15 mL/min), renal replacement therapy, or history of organ transplant. The NAFLD fibrosis and FIB-4 scores have good negative predictive values and can be used clinically to exclude advanced fibrosis in patients who have low scores [16]. The study therefore selectively recruited patients who were more likely to have clinically significant liver disease.

### 2.2. Study Population

Eligible patients were invited to attend the liver clinic at the Princess Alexandra Hospital, Brisbane, for clinical assessment. Informed written consent was obtained from each patient and the protocol was approved by the Metro South Health and the University of Queensland Human Research Ethics Committees (HREC/15/QPAH/301; UQ2015001047).

### 2.3. Clinical Data

Data were collected prospectively by the study clinician (PP). Firstly, medical history was obtained during a consultation in the liver clinic. Patients underwent a clinical assessment and were subsequently informed about their diagnosis.

Medical history was obtained using a structured questionnaire. Interview items included self-reported sociodemographic characteristics, history of tobacco or recreational drug use, previously diagnosed liver disease, other medical conditions, and use of medications. Alcohol intake was assessed using a standardized questionnaire and the Alcohol Use Disorders Identification Test [17] (AUDIT). The AUDIT was developed by the World Health Organization and is the most widely used measure of alcohol consumption and alcohol-related problems. The AUDIT-C, consisting of the first three AUDIT questions (how often is alcohol consumed, how many alcoholic drinks are typically drunk in a day, and how often are six or more drinks consumed), provides a standardized continuous measure of alcohol consumption. Alcohol intake within the preceding 5 years was used to define NAFLD, and drinkers consumed <20 g/day. Lifetime drinking history classified patients into nondrinkers (never drinkers), light drinkers (always ≤20 g/day), and moderate drinkers (any period with intake >20 g/day).

Clinical assessment included anthropometric measurements (weight, height, and waist circumference), laboratory tests (routine biochemical, hematological, and serological assays), transient elastography, and liver ultrasound. Metabolic syndrome was defined as central obesity (waist circumference: Europid male ≥94 cm, South Asian male ≥90 cm, female ≥80 cm), plus any two of the following four factors: raised fasting plasma glucose or previously diagnosed type 2 diabetes, raised blood pressure or treatment of previously diagnosed hypertension, raised triglycerides or reduced HDL cholesterol, or specific treatment for these lipid abnormalities [18].

Transient elastography with simultaneous Controlled Attenuation Parameter (CAP) was performed after a 3-hour fast using FibroScan® technology (Echosens, Paris, France) using the standard M or XL probes. Examinations were performed by a trained clinical nurse (with experience in performing more than 250 liver stiffness measurements (LSM)) and reviewed by a hepatologist (KAS) with extensive FibroScan experience (more than 2000 LSM performed). Recommended standard FibroScan operating procedures were followed along with adherence to criteria for definition of reliable LSM: minimum of 10 valid measurements with a success rate of ≥60% and IQR ≤30% of the final (median) result. The XL probe was used when the skin-capsule depth was ≥2.5 cm. Although optimal liver stiffness cut-off values in NAFLD remain under discussion, for the purposes of this study, we used the following cut-off values: 8.2 kPa for severe fibrosis and 10.5 kPa for the diagnosis of cirrhosis, respectively, for* ruling out* disease with at least 90% sensitivity, as described by Cassinotto et al. [19].

Liver ultrasound was performed by one of the sonographers at the Department of Diagnostic Radiology, Princess Alexandra Hospital, Brisbane, trained in advanced liver imaging. The Princess Alexandra Hospital is the referral center for the state-wide liver transplant service, and over 1650 liver ultrasounds are performed in the department each year. Steatosis was determined by increased hepatic echogenicity and beam attenuation, resulting in the renal cortex appearing relatively hypoechoic to the liver parenchyma, absence of the normal echogenic walls of the portal vein, and poor visualization of the diaphragm and deep portions of the liver [20]. Evidence of cirrhosis or portal hypertension on liver imaging was determined by liver surface nodularity or signs of portal hypertension including portal vein dilatation, splenomegaly, portosystemic collaterals, and ascites.

NAFLD severity was broadly classified by transient elastography and liver ultrasound. Participants were categorized as “no advanced disease” (LSM < 8.2 kPa and no evidence of cirrhosis or portal hypertension on liver imaging) and “clinically significant liver disease” (LSM ≥ 8.2 kPa). Liver biopsy was performed in a subset of the patients (*n* = 28) for clinical indications, based on increased likelihood of advanced disease or patient interest in participating in clinical therapy trials. Histological changes of NAFLD (steatosis, lobular and portal inflammation, presence of ballooning, Mallory-Denk bodies, and fibrosis) were assessed and scored according to accepted criteria [21, 22].

### 2.4. Data Analysis

Data analysis was conducted using SPSS (SPSS Inc.) version 24.0 (College Station, TX; StataCorp LP, 2013). Participants' sociodemographic and clinical characteristics were described using frequency distribution and percentage and mean and standard deviation (data normally distributed). Pearson's chi-square tests were used to compare proportions (Fisher's exact test was used when cell counts were less than 5) and one-way ANOVA was used to compare means between groups. All* p* values were 2-sided and statistical significance was set at alpha = 0.05. Multinomial logistic regression was used to calculate adjusted *p* values between nondrinkers, light drinkers, and moderate drinkers.

Bivariable logistic regression analysis was used to determine the odds of being categorized as having “clinically significant liver disease” compared to “no advanced disease.” Obesity or girth, age, and gender, as variables of clinical relevance, were included in the model. Odds ratios (OR) and 95% confidence interval (CI) were reported.

## 3. Results

### 3.1. Study Population and Alcohol Consumption

One hundred and fifty-one “at-risk” patients with T2DM and NAFLD were reviewed in the NAFLD clinic over a 12-month period. Overall, the mean age of subjects was 61 ± 10.3 years and 63.6% were males. The majority of subjects were Caucasian, with a mean body mass index (BMI) of 35.9 ± 8.3 kg/m^2^ and mean girth of 120.8 ± 18.6 cm. The cohort included 46 lifetime nondrinkers, 70 light drinkers (never more than 20 g alcohol/day), and 35 moderate drinkers (any prior period of alcohol intake >20 g/day). Moderate drinkers had significantly higher median lifetime alcohol consumption of 66.7 g/week compared with light drinkers (4.8 g/week) and nondrinkers (0 g/week) (*p* = 0.000) and had a mean of 23.7 (±10.9) years from their period of regular drinking >20 g/day up to the time of enrolment in the study. In validation of the alcohol history-based subgrouping, moderate drinkers had a significantly higher alcohol consumption score and alcohol-related harm score (4) when compared with light drinkers (2) and nondrinkers (0) (*p* = 0.012 and 0.000, resp.) (Supplementary Table 1 in Supplementary Material available online at https://doi.org/10.1155/2017/7927685). A post hoc analysis demonstrated a significant difference between moderate and light drinkers' alcohol consumption (*p* = 0.035), further corroborating the alcohol history taken at the time of interview.

### 3.2. Alcohol Consumption and Clinical/Metabolic Characteristics

The demographic and clinical characteristics of the 151 subjects according to lifetime drinking history are summarized in Table 1. Compared with lifetime nondrinkers, light and moderate drinkers were more likely to be male (*p* = 0.008) and Caucasian (*p* = 0.007) and to have a history of cigarette smoking (*p* = 0.000). Girth and BMI were not significantly different between the three cohorts (*p* > 0.05). Although there was no significant difference in glycated hemoglobin (HbA1c) or insulin therapy between the three cohorts, significantly more moderate drinkers required ≥3 hypoglycemic agents to maintain diabetic control (*p* = 0.041), suggesting that moderate drinkers may have worse diabetic control. In particular, a higher proportion of moderate drinkers (49%) were using a glucagon-like peptide type 1 (GLP-1) receptor agonist or a dipeptidyl peptidase-4 (DPP-4) inhibitor, compared to light drinkers (26%) and nondrinkers (20%) (*p* = 0.027).

Serum triglyceride levels were higher in moderate drinkers (*p* = 0.033) but this was not statistically significant following adjustment for age and BMI (*p* = 0.086). Importantly, a higher proportion of moderate drinkers (20%) were taking a fibrate medication to lower blood triglyceride levels, compared to light drinkers (4%) and nondrinkers (11%) (*p* = 0.044). Serum low-density lipoprotein (LDL) levels were significantly lower in females with a history of moderate alcohol intake (*p* = 0.045); however, this should be interpreted with caution as the number of females in this subgroup was very small (*n* = 7) (data not presented). There was no significant difference in HDL levels or use of statin therapy between the three cohorts (*p* > 0.05).

Interestingly, the prevalence of obstructive sleep apnea (OSA) differed significantly between patients with and without alcohol consumption (*p* = 0.003). Seventy-one percent of moderate drinkers had a history of obstructive sleep apnea compared to 36% of light drinkers and 17% of nondrinkers. Self-reported depression was also significantly more common in moderate drinkers (71%), compared to light and nondrinkers (35% and 48%, resp.; *p* = 0.003).

### 3.3. Alcohol Consumption and NAFLD Fibrosis Severity

LSM met quality criteria in 134 patients. Median LSM was 6.3 kPa with a range from 2.6 to 63.9 kPa and required the use of the XL probe in 83%. Eighty-eight of 134 patients (65.7% of the cohort) had LSM <8.2 kPa, suggesting the absence of severe fibrosis. Liver biopsy was performed in a subset of the patients (*n* = 28), and an additional 8 patients had liver imaging diagnostic of cirrhosis. Twenty-nine of these 36 patients had liver stiffness measurements ≥8.2 kPa and 23 of these 29 patients (79%) had bridging fibrosis or cirrhosis.

There was no significant difference between those patients with LSM <8.2 kPa or ≥8.2 kPa and their alcohol consumption, dependence, alcohol-related harm, and overall AUDIT score. Compared to male lifetime nondrinkers, the proportion of male light and moderate drinkers with LSM ≥8.2 kPa (24, 35 and 37 per cent, resp.) was not significantly different (*p* > 0.05). In females, the proportion of patients with LSM ≥ 8.2 kPa was 35% in nondrinkers and 50% in light drinkers. However, none of the 7 female patients with a history of moderate alcohol intake had LSM ≥ 8.2 kPa. Compared to lifetime nondrinkers, light drinkers had 1.79 (95% CI: 0.67–4.82; *p* = 0.247) and moderate drinkers had 0.91 (95% CI: 0.27–3.10; *p* = 0.881) times the odds of having LSM ≥ 8.2 (adjusted for age, gender, and BMI).

Similarly, there was no significant difference in biochemical, hematological, or liver imaging parameters between the three patient groups (Table 2).

## 4. Discussion

Increasing evidence suggests that moderate alcohol consumption (≤30 g/day) reduces the risk of coronary artery disease and other vascular conditions associated with the metabolic syndrome [23–27]. However, the effect of modest alcohol intake on the* liver* component of the metabolic syndrome (NAFLD) and other comorbid conditions remains controversial. Due to the high prevalence of NAFLD and increased risk of advanced fibrosis in people with T2DM, determining the effect of light or moderate alcohol intake on liver disease progression in these patients is crucial. The present study indicated that, in diabetic patients at risk of clinically significant NAFLD, light or moderate lifetime alcohol consumption was not associated with a protective effect on liver fibrosis. Of concern, alcohol consumption was associated with an increased prevalence of metabolic comorbidities, specifically hypertriglyceridemia and OSA, and a history of smoking and self-reported depression.

Prior studies examining the relationship between alcohol, obesity, and liver injury have produced conflicting results. In comparison with obesity alone, an earlier cross-sectional study showed that the combination of obesity and heavy alcohol intake (>60 g/day) was associated with a 1.3-fold increased risk for steatosis detected by liver ultrasound [28]. The effects of alcohol intake and body weight on liver enzyme levels were examined in a large US population-based study [29]. Among obese and overweight subjects, alcohol intake of ≥1 and >2 standard drinks per day, respectively, was associated with a higher prevalence of elevated aminotransferase levels in multivariate analyses [29]. In contrast to these data, a number of studies have suggested that low-to-moderate alcohol intake may be associated with a lower risk of obesity-related liver disease [30], possibly due to the beneficial effect of reduced insulin resistance [31]. Among severely obese patients (BMI > 35 kg/m^2^) presenting for weight loss surgery, modest alcohol consumption (<200 g/week) was associated with a reduction in NASH, but the effect was not significant after controlling for diabetes or insulin resistance index [31]. In comparison with no alcohol use, self-reported wine consumption up to 10 g/day was associated with a lower prevalence of NAFLD (defined using unexplained serum alanine aminotransferase (ALT) elevation) among participants in the Third National Health and Nutrition Examination Survey [30].

Few studies have examined the effect of modest alcohol consumption in the presence of preexisting NAFLD. Heavy episodic drinking (>60 or 48 g ethanol in males and females, resp., consumed on one occasion at least once a month) was associated with fibrosis progression on repeat liver biopsy or development of end-stage liver disease after a mean of 13.8 years [12]. In contrast, light alcohol consumption (up to 2 drinks per day) was associated with lesser degrees of NAFLD severity in three cross-sectional studies [9–11]. However, in these studies, the prevalence of patients with T2DM or metabolic syndrome was relatively low, ranging from 7% to 41% of the study populations [10–12, 32]. In our cohort of diabetic subjects, light or moderate alcohol consumption was not associated with lesser degrees of NAFLD severity as assessed by liver stiffness, a noninvasive marker of liver fibrosis. Reasons for the lack of liver disease protection in our patient cohort are unclear but may include alcohol-related exacerbation of lipotoxicity, liver inflammation, and oxidative stress in the presence of type 2 diabetes [33].

In our study, fasting serum triglyceride levels were higher in moderate drinkers, and a higher proportion of moderate drinkers (20%) were taking a fibrate medication to lower blood triglyceride levels, compared to light drinkers (4%) and nondrinkers (11%). Although high alcohol intake is known to increase blood triglyceride levels, prior studies of the effect of low-to-moderate alcohol intake on triglycerides have provided variable results [34]. As alcohol can impair lipolysis and raise very-low-density lipoprotein (VLDL) levels [35], alcohol intake in diabetic patients may worsen their predisposition to hypertriglyceridemia. This is a consequence of raised VLDL and increased cholesteryl ester transfer protein activity causing a higher hepatic flow of free fatty acids in combination with a deficiency in lipoprotein lipase activity [35]. Although currently there remain no consensus guidelines regarding appropriate triglyceride levels, this association is of concern as evidence suggests that hypertriglyceridemia independently increases the risk of cardiovascular events [36–38].

We have previously shown a high burden of multimorbidity in diabetic NAFLD patients, with the most common coexisting chronic conditions including ischemic heart disease, obstructive sleep apnea, self-reported depression, respiratory diseases, and osteoarthritis [39]. Our data also demonstrated an association between OSA and alcohol consumption: 71% of moderate drinkers had a history of OSA compared to 36% of light drinkers and 17% of nondrinkers. This is consistent with prior studies demonstrating an association between alcohol use and OSA [40]. Alcohol has been shown to relax the upper airway whilst diminishing the normal arousal response to airway obstruction, resulting in impaired normal breathing with sleep fragmentation and sleep disordered breathing [40]. Similarly, self-reported depression was very prevalent in this patient cohort and was significantly more common in moderate drinkers compared to light and nondrinkers. The high prevalence of depression has been corroborated by a recent meta-analysis, where 30% of T2DM patients reported depression [41]. In particular, elderly patients suffering from depression and alcohol misuse have worse health outcomes, poorer physical and mental health, and a higher mortality rate [42]. Alcohol abuse can also result in unsatisfactory patient control of diabetes and hypertension [42].

Study strengths include the prospective collection of history of alcohol consumption by the study clinician before a diagnosis was established and face-to-face patient interviews to overcome literacy concerns and patient understanding of questions. The downside of using face-to-face interviews is that they may promote social-desirability bias, where patients answer questions in a certain way or feel self-conscious in the presence of an interviewer, thereby inaccurately or incompletely disclosing alcohol consumption. This was a small single-center study and therefore our findings may not be truly representative of the wider population with T2DM and NAFLD. In order to ensure we were assessing the impact of alcohol intake in patients with NAFLD, participants with alcohol intake >20 g/day within 5 years prior to study entry were excluded. However, these exclusion criteria may have led to a selection bias excluding patients in whom moderate ongoing alcohol intake may have been beneficial, resulting in resolution of NAFLD. Although liver biopsy remains the gold standard for the diagnosis and assessment of liver fibrosis, the method of assessing advanced fibrosis used in this study (LSM) is an accepted noninvasive procedure for identification of advanced fibrosis [43].

## 5. Conclusion

Lifetime alcohol consumption was not significantly associated with liver fibrosis in T2DM patients when assessed by LSM. Of concern, alcohol consumption was associated with an increased prevalence of metabolic comorbidities, specifically hypertriglyceridemia and OSA, and a history of smoking and self-reported depression. This study highlights the importance of taking a detailed alcohol history from patients regarded as “without a history of significant alcohol consumption,” as the impact of alcohol on diabetes comorbidities cannot be dismissed and requires further investigation.

## Supplementary Material

Supplementary table 1: AUDIT questionnaire breakdown according to lifetime drinking history.

## Figures and Tables

**Table 1:** Demographic and clinical characteristics of the 151 subjects according to lifetime drinking history.

	Nondrinker (*n* = 46)	Light drinker (*n* = 70)	Moderate drinker (*n* = 35)	Crude *p* values	Adjusted *p* values^b^
*Age*	62.8 ± 10.6	61.2 ± 9.9	58.3 ± 10.4	0.147	0.167
*Gender (male)* ^*a*^	22 (48)	46 (66)	28 (80)	**0.010**	**0.008**
*Ethnicity (Caucasian)* ^*a*^	28 (61)	58 (83)	30 (86)	**0.008**	**0.007**
*BMI (kg/m* ^*2*^)					
Male	33.1 ± 7.5	34.5 ± 6.4	36.4 ± 0.2	0.170	0.313^c^
Female	37.3 ± 10.3	39.1 ± 9.1	36.4 ± 8.6	0.519	0.313^c^
*Girth (cm)*					
Male	115.5 ± 16.3	120.4 ± 16.5	126.2 ± 18.7	0.098	0.173^c^
Female	119.3 ± 20.1	121.7 ± 17.7	111.6 ± 24.9	0.338	0.204^c^
*Never smoker* ^*a*^	36 (78)	30 (43)	13 (37)	**0.000**	**0.000**
*Diabetic “control”*					
HbA1c	7.9 ± 1.3	8.0 ± 1.7	8.5 ± 1.6	0.197	0.515
Insulin therapy^a^	23 (50)	32 (46)	22 (63)	0.250	0.239
DPP4/GLP1^a^	9 (20)	18 (26)	17 (49)	**0.012**	**0.027**
*Dyslipidemia*					
Triglycerides	2.0 ± 1.5	2.0 ± 1.1	2.8 ± 2.1	**0.033**	0.086
Fibrate therapy^d^	5 (11)	3 (4)	7 (20)	**0.037**	**0.044**
*Comorbidities*					
Ischemic heart disease^a^	10 (22)	20 (29)	13 (37)	0.314	0.300
Obstructive sleep apnea^a^	8 (17)	25 (36)	25 (71)	**0.005**	**0.003**
Depression^a^	22 (48)	25 (35)	25 (71)	**0.003**	**0.003**
Respiratory disease^a^	9 (20)	15 (21)	10 (29)	0.603	0.615
Osteoarthritis^a^	15 (33)	29 (41)	13 (37)	0.629	0.487

Data presented continuously (mean ± standard deviation) and analyzed using one-way ANOVA, unless specified. ^a^Data presented categorically (*n*, %) and analyzed using Pearson's *χ*^2^ test. ^b^All *p* values adjusted for age and BMI unless specified. ^c^Adjusted for age. ^d^Data presented categorically (*n*, %) and analyzed using Fisher's exact test.

**Table 2:** Liver stiffness measurements and selected biochemical, hematological, and liver imaging parameters according to lifetime drinking history.

	Nondrinker (*n* = 46)	Light drinker (*n* = 70)	Moderate drinker (*n* = 35)	Crude *p* value	Adjusted *p* value^e^
*LSM kPa* ^*a*^					
All	8.4 ± 8.0	9.8 ± 7.1	10.7 ± 12.5	0.570	0.546
Male	8.7 ± 10.1	8.0 ± 4.8	12.0 ± 13.4	0.226	0.639
Female	8.2 ± 5.1	12.4 ± 9.8	4.6 ± 1.7	0.053	**0.016**
*LSM ≥ 8.2 kPa* ^*a*^					
All	12 (29)	24 (40)	10 (30)	0.459	0.302
Male	5 (24)	14 (35)	10 (37)	0.584	0.581
Female^b^	7 (35)	10 (50)	0 (0)	0.092	0.132
*CAP (dB/m)* ^*a*^	322.1 ± 68.5	333.4 ± 54.2	340.6 ± 59.5	0.910	0.492
*ALT (IU/ml)*	36.4 ± 26.4	38.4 ± 29.5	37.4 ± 15.8	0.909	0.818
*AST (IU/ml)*	29.0 ± 17.6	28.6 ± 26.1	24.7 ± 11.2	0.606	0.457
*GGT (IU/ml)*	44.2 ± 42.9	53.6 ± 83.1	47.6 ± 45.4	0.738	0.750
*Platelets (×10* ^*9*^)	242.0 ± 62.6	229.5 ± 58.7	255.0 ± 45.6	0.095	0.081
*Albumin (g/dL)*	40.4 ± 2.8	40.6 ± 2.7	41.0 ± 3.4	0.704	0.601
*Spleen size (cm)* ^*c*^	10.6 ± 2.9	10.4 ± 2.9	9.9 ± 3.1	0.666	0.617
*Liver size (cm)* ^*d*^	17.3 ± 3.5	17.0 ± 2.3	17.9 ± 3.4	0.501	0.502

Data presented continuously (mean ± standard deviation) and analyzed using one-way ANOVA. Data presented categorically (*n*, %) and analyzed using Pearson's *χ*^2^ test, unless specified. ^a^Smaller cohort of patients as LSM met quality criteria in 134 patients. ^b^Data presented categorically (*n*,%) and analyzed using Fisher's exact test.^c^Spleen size documented in 99 patients. ^d^Liver size documented in 94 patients.^e^All *p* values adjusted for age and BMI unless specified. ALT: alanine aminotransferase; AST: aspartate aminotransferase; GGT: gamma-glutamyl transferase.

## Abbreviations

NAFLD: Nonalcoholic fatty liver disease
T2DM: Type 2 diabetes mellitus
HDL: High-density lipoprotein
NASH: Nonalcoholic steatohepatitis
AUDIT: Alcohol Use Disorders Identification Test
CAP: Controlled Attenuation Parameter
LSM: Liver stiffness measurement
OR: Odds ratios
CI: Confidence interval
BMI: Body mass index
HbA1c: Glycated hemoglobin
GLP-1: Glucagon-like peptide type 1
DPP-4: Dipeptidyl peptidase-4
LDL: Low-density lipoprotein
OSA: Obstructive sleep apnea
ALT: Alanine aminotransferase
VLDL: Very-low-density lipoprotein
AST: Aspartate aminotransferase
GGT: Gamma-glutamyl transferase.
