# Supplementary material for: Alcohol Consumption in Diabetic Patients with Nonalcoholic Fatty Liver Disease
Source: Can J Gastroenterol Hepatol. 2017 Nov 1;2017:7927685. doi: 10.1155/2017/7927685 (PMC5687130; doi:10.1155/2017/7927685)
Supplement: Supplementary file 1 — Supplementary table 1: AUDIT questionnaire breakdown according to lifetime drinking history. [file 7927685.f1.pdf]

Supplementary Table 1: AUDIT questionnaire breakdown according to lifetime drinking history.

	Non drinker	Light drinker	Moderate Drinker	Crude P Values
**Consumption score <6**	46 (100)	70 (100)	32 (91.4)	0.012
**Dependence Score <4**	46 (100)	70 (100)	35 (100)	1.000 [a]
**Alcohol related harm score <1**	46 (100)	60 (85.7)	15 (42.9)	0.000
**AUDIT score < 7**	46 (100)	69 (98.6)	29 (82.9)	0.001

Data presented categorically (n, %) and analysed using a Pearson χ^2 test, unless specified; [a] Data presented categorically n (%) and analysed using Fisher's exact test.
